# The blue fluorescent protein from *Vibrio vulnificus* CKM-1 is a useful reporter for plant research

**DOI:** 10.1186/s40529-014-0079-x

**Published:** 2014-12-17

**Authors:** Jin-Min Tu, Ming-Chung Chang, Lynn LH Huang, Ching-Dong Chang, Hao-Jen Huang, Ruey-Hua Lee, Ching-Chun Chang

**Affiliations:** 1 Institute of Tropical Plant Sciences, National Cheng Kung University, 1 University Rd, Tainan, 701 Taiwan; 2 Department of Nutrition, Hung Kuang University, Taichung, 433 Taiwan; 3 Institute of Biotechnology, National Cheng Kung University, Tainan, 701 Taiwan; 4 Department of Veterinary Medicine, National Pingtung University of Science and Technology, Pingtung, 912 Taiwan; 5 Department of Life Sciences, National Cheng Kung University, Tainan, 701 Taiwan

**Keywords:** Reporter, Blue fluorescence protein, NADPH, Vibrio vulnificus CKM-1

## Abstract

**Background:**

The mBFP is an improved variant of NADPH-dependent blue fluorescent protein that was originally identified from the non-bioluminescent pathogenic bacteria *Vibrio vulnificus* CKM-1. To explore the application of mBFP in plants, the mBFP gene expression was driven by one of the three promoters, namely, leaf-specific (*RbcS*), hypoxia-inducible (*Adh*) or auxin-inducible (*DR5*) promoters, in different plant tissues such as leaves, roots and flowers under diverse treatments. In addition, the expressed mBFP protein was targeted to five subcellular compartments such as cytosol, endoplasmic reticulum, apoplast, chloroplast and mitochondria, respectively, in plant cells.

**Results:**

When the mBFP was transiently expressed in the tobacco leaves and floral tissues of moth orchid, the cytosol and apoplast exhibited brighter blue fluorescence than other compartments. The recombinant mBFP-mS_1_C fusion protein exhibited enhanced fluorescence intensity that was correlated with more abundant RNA transcripts (1.8 fold) as compared with a control. In the root tips of horizontally grown transgenic Arabidopsis, mBFP could be induced as a reporter under hypoxia condition. Furthermore, the mBFP was localized to the expected subcellular compartments, except that dual targeting was found when the mBFP was fused with the mitochondria-targeting signal peptide. Additionally, the brightness of mBFP blue fluorescence was correlated with NADPH concentration.

**Conclusion:**

The NADPH-dependent blue fluorescent protein could serve as a useful reporter in plants under aerobic or hypoxic condition. However, to avoid masking the mitochondrial targeting signal, fusing mBFP as a fusion tag in the C-terminal will be better when the mBFP is applied in mitochondria trafficking study. Furthermore, mBFP might have the potential to be further adopted as a NADPH biosensor in plant cells. Future codon optimization of mBFP for plants could significantly enhance its brightness and expand its potential applications.

**Electronic supplementary material:**

The online version of this article (doi:10.1186/s40529-014-0079-x) contains supplementary material, which is available to authorized users.

## Background

Fluorescent proteins have been widely used as specific markers to study gene and protein behaviors at the cellular, tissue, and organismal levels. The green fluorescent protein (GFP) from *Aequorea victoria* is the best studied and most widely used, and many other spectral variants have been derived from modification made to the GFP (Ai et al. [2007]; Cubitt et al. [1995]; Shaner et al. [2005]). Previously, blue fluorescent proteins (BFPs) derived from GFP were engineered by modifying the chromophore structures through the substitution of Tyr^66^ with a histidine (Ai et al. [2007]; Heim and Tsien [1996]; Lippincott-Schwartz and Patterson [2003]). However, several drawbacks of BFP variants that might limit their application have been reported, including low solubility, poor photostability, slow maturation times, and low quantum yields (Heim and Tsien [1996]; Lippincott-Schwartz and Patterson [2003]). Recently, the BFPs variants have been greatly improved which make them applicable in the imaging of mammalian cells (Ai et al. [2007]; Mena et al. [2006]; Subach et al. [2008], [2011]). For instance, Azurite, a BFP variant which is significantly enhanced in photostability and brightness (Mena et al. [2006]). Subsequently, several BFP derivatives that are even brighter than azurite, such as mKalama1, EBFP1.5, and EBFP2, were obtained, but only EBFP2 has better photostability. However, mKalama1 has a brighter fluorescence than EBFP2 due to the high efficiency in folding and chromophore maturation (Ai et al. [2007]). Alternatively, mTagBFP, a red fluorescent protein (RFP) derivative carrying a tyrosine-based chromophore has substantially higher brightness, faster chromophore maturation, and higher pH stability than BFPs with a histidine in the chromophore (Subach et al. [2008]). Meanwhile, mTagBFP2 exhibits greater chemical stability and photostability and substantially higher brightness in live cells, while maintaining the same other beneficial properties as mTagBFP (Subach et al. [2011]). However, BFPs and other GFP variants are not easily applicable in real-time imaging under anaerobic conditions due to the formation of chromophore through the oxidation of amino acid residues (Shaner et al. [2005]).

The oxygen-independent blue fluorescent proteins belonging to the short-chain dehydrogenase (SDR) family that have been identified either from a bacterial pathogen (Su et al. [2001]) or more recently from metagenomic DNA (Hwang et al. [2012]) might provide a alternative source for imaging live cells under both aerobic and anaerobic conditions. Previously, the NADPH-dependent blue fluorescent protein (BFPvv) gene was originally identified from the non-bioluminescent pathogen *Vibrio vulnificus* CKM-1 (Su et al. [2001]). Subsequently, random mutagenesis and DNA shuffling approaches have been applied to greatly increase the intensity and duration of BFPvv blue fluorescence (Su et al. [2001]; Chang et al. [2004a]; Kao et al. [2011]). The mechanism of the blue light emission from BFPvv is based on effectively augmenting the intrinsic fluorescence of bound NADPH (Chang et al. [2004b]; Kao et al. [2011]). The crystal structure of the BFPvvD8-NADPH complex has revealed that the increased fluorescent intensity of BFPvv is related to the conformational change caused by a Gly^176^ mutation close to the NADPH binding site (Kao et al. [2011]).

Due to the potential interference from auto-emission of blue fluorescence through the cinnamic acid covalently bound with the cell wall and other minor phenolic compounds present in the plant cell wall (Buschmann et al. [2000]), the BFPs of GFP variants have been rarely applied in plant research. To our best knowledge, the only previous study in addressing the application of BFP, a Y66H type of GFP variants have shown limited utility in monitoring viral movement in plants because its brightness was significantly influenced by the cellular pH environments among plant species (Diveki et al. [2002]). In this study, we explored the possibilities for the application of NADPH-dependent BFPvv from *Vibrio vulnificus* CKM-1 in plants. By expressing the improved variant of BFPvv in different subcellular compartments and tissue types among plant species, our results demonstrated that the BFPvv variant is a useful reporter for plant research.

## Methods

### Construction of plant nuclear expression plasmids

**Figure 1:**
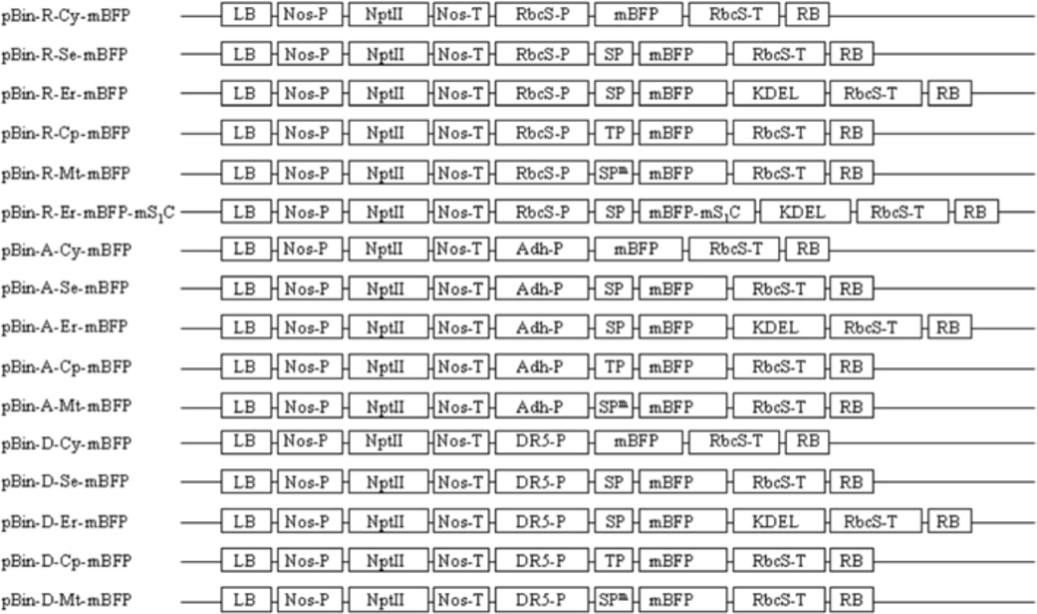
**Schematic representation of plant expression vectors.** The mBFP or mBFP-mS_1_C fusion gene expression cassettes driven by three different promoters in pBINPLUS vector were shown. Nos-P, *Nos* promoter; Nos-T, *Nos* terminator; NptII, Kanamycin resistance gene; RbcS-P, *RbcS* promoter; RbcS-T, *RbcS* terminator; Adh-P, *Adh* promoter; DR5-P, DR5 promoter; mBFP, variants of blue fluorescence protein (BFPvv) gene; mBFP-mS_1_C, fusion of mBFP gene with 3′ end region of ARV *S1* gene; KDEL, ER retention signal; SP, signal peptide of secretory pathway; SP^m^, mitochondrial signal peptide; TP, chloroplast transit peptide.

The mBFP gene which was genetically modified variant of BFPvv from *Vibrio vulnificus* CKM-1 was PCR amplified and cloned into five different plant expression vectors of ImpactVector series (Wageningen UR, Netherlands). Additionally, the mBFP-mS_1_C fusion gene, in which the mBFP gene was fused with the 3′ end region (528 bp) of the avian reovirus (ARV) *S1* gene (Lu et al. [2011]), was cloned into ImpactVector. In addition, the *Adh* promoter was PCR amplified from *Arabidopsis thaliana* ecotype Columbia according to a previous study (McKendree and Ferl [1992]), and was used to replace the *RbcS* promoter in the mBFP expression cassettes. Furthermore, the auxin-inducible DR5 promoter was synthesized (MDbio, Taiwan) according to a previous report (Ulmasov et al. [1997]), and was also used to replace the *RbcS* promoter in the mBFP expression cassettes. Subsequently, the mBFP or mBFP-mS_1_C fusion gene expression cassettes driven by three different promoters in the ImpactVector-based plasmids were pasted into the PacI/AscI site of the pBINPLUS vector (Figure 1). To make sure no mutations were introduced during above PCR cloning, the newly constructed plasmids were sequenced. The PCR primers used in the cloning was listed in Additional file 1: Table S1. The plant expression vectors were transformed into *Agrobacteria tumerfaciens* GV3101 by electroporation.

### Agroinfiltration of tobacco and moth orchid

The agroinfiltration procedure was performed according to a previous study (Sparkes et al. [2006]). The *Agrobacteria* carried with or without expression constructs were used to infiltrate tobacco (*Nicotiana tabacum* cv. Petit Havana) leaves and the petals of moth orchid (*Phalaenopsis aphrodite* subsp. *formosana*). The infiltrated plants were kept in dark for 2 days and then under light for 3 days except as indicated otherwise. The infiltrated tissues were treated with or without 50 μM NAA for 24 hr. Alternatively, the infiltrated floral tissues of moth orchid were treated with or without 1 mM H_2_O_2_ for 24 hr. The tissues were imaged under a fluorescence microscope (Olympus BX53, Japan) equipped with a UV light with an excitation wavelength of 330 ~ 385 nm and a filter with an emission wavelength of 420 nm. The images were captured by the Olympus U-TV1x-2 camera system under the control of SPOT imaging software (Spot Imaging Solutions, USA).

### Transgenic Arabidopsis and protoplast preparation

The *Agrobacteria tumerfaciences* GV3101 carrying expression construct was used to transform *Arabidopsis thaliana* ecotype Columbia by floral dip method (Zhang et al. [2006]). The T_0_ transgenic plants were selected in MS media containing kanamycin (50 μg/ml) until T_2_ or T_3_ generation as indicated. The integration of foreign DNA into nuclear genome of transgenic Arabidopsis was checked by PCR using KAPA3G plant PCR kits (Antec Bioscience, Taiwan) according to manufacturer’s procedure. The seeds of transgenic Arabidopsis lines were plated onto the MS media containing kanamycin (50 μg/ml) and then grown horizontally or vertically in a growth chamber with 8/16 hr of dark/light cycle at 22°C. The protoplast preparation was referred to a previous report (Yoo et al. [2007]). In brief, the leaves of transgenic Arabidopsis or Agrobacteria-infiltrated tobacco leaves were sliced into about 0.5 mm segments and immersed into enzyme solution containing macerozyme R10 (Duchefa, Netherlands) and cellulose R10 (Yakult Honsha, Japan). After washing, the protoplasts were imaged under a confocal microscope (Zeiss LSM780).

### RT-PCR assay

Total RNA was isolated from infiltrated tobacco leaves by using a total RNA mini purification kit (BioKit, Taiwan) according to the manufacturer’s procedure. After removing any trace amounts of DNA contamination, the RNA (1 μg) was reverse transcribed into cDNA with oligo-dT as the primer using a cDNA synthesis kit (Bioline, UK) according to the manufacturer’s protocol. Subsequently, the gene-specific primers of mBFP and tobacco elongation factor 1α (*Nt* EF-1α) listed in Additional file 1: Table S1 were used to carry out PCR amplification with cDNA as template. The PCR condition was according to a previous study (Lu et al. [2011]), except 28 cycles was carried out. The PCR products were electrophoresed on a 0.8% agarose gel and then visualized by staining with ethidium bromide. The relative abundance of mBFP RT-PCR products to that of *Nt* EF-1α was further quantitated by Image J (National Institutes of Health, USA).

### NADPH treatment

The leaves of 4-week old wild type (WT) and transgenic Arabidopsis pBin-R-Se-mBFP line or pBin-R-Cy-mBFP line (Figure 1) were directly infiltrated with different concentrations of NADPH solution as indicated and kept at room temperature for 5 min before imaging. Alternatively, the protoplasts were isolated from infiltrated tobacco leaves, and incubated with 0, 0.25 or 0.5 μM NADPH in final concentration for 5 min, and subsequently the protoplasts were imaged using a confocal microscope.

## Results and discussion

### Construction of plant expression vectors

**Figure 2:**
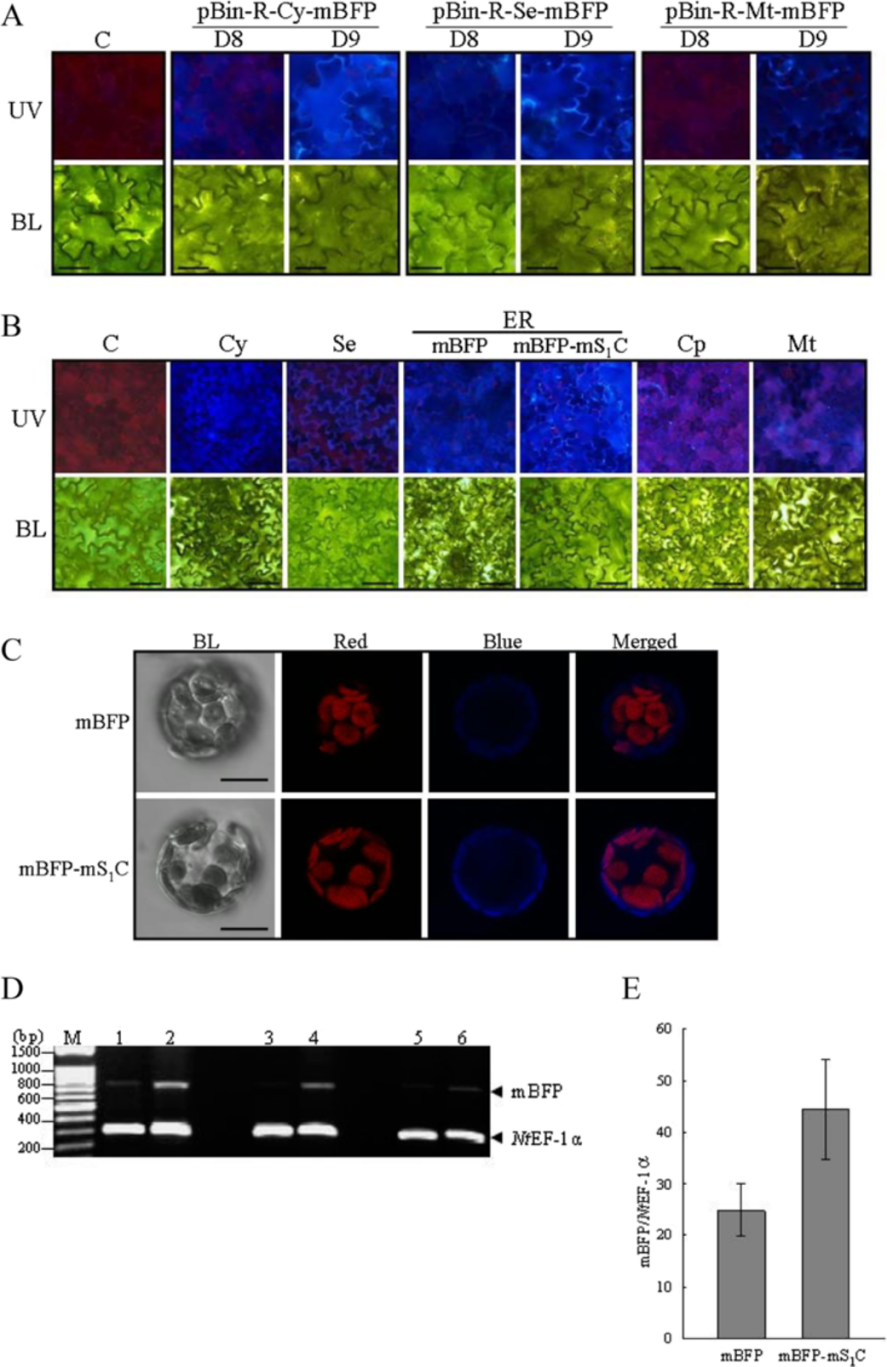
**Transient expression of mBFP and mBFP-mS**_**1**_**C fusion protein in tobacco leaves by agroinfiltration. A**. The mBFPvvD8 (D8) or mBFPvvD9 (D9) gene expression constructs driven by *RbcS* promoter were transiently expressed in tobacco leaves by agroinfiltration, respectively. The infiltrated tissues were imaged under fluorescence microscope with UV excitation (UV) or bright light (BL). C, *Agrobacteria* only as control. The magnification is 400 X and the ruler is 20 μm. **B**. The mBFP or mBFP-mS_1_C fusion gene expression cassettes driven by *RbcS* promoter were transiently expressed in tobacco leaves by agroinfiltration, respectively, and the proteins were targeted to cytosol (Cy, pBin-R-Cy-mBFP), apoplast (Se, pBin-R-Se-mBFP), ER (mBFP, pBin-R-Er-mBFP; mBFP-mS_1_C, pBin-R-Er-mBFP-mS_1_C), chloroplast (Cp, pBin-R-Cp-mBFP) or mitochondria (Mt, pBin-R-Mt-mBFP), respectively. The infiltrated tissues were imaged under fluorescence microscope with UV excitation (UV) or bright light (BL). C, *Agrobacteria* only as control. The magnification is 200 X and the ruler is 40 μm. **C**. The protoplasts were isolated from tobacco leaves that transiently expressed pBin-R-Er-mBFP (mBFP) or pBin-R-Er-mBFP-mS_1_C (mBFP-mS_1_C) constructs, and were imaged by confocal microscope. BL, bright light; Red, chlorophyll auto-fluorescence; Blue, mBFP blue fluorescence. The magnification is 1,000 X and the ruler is 20 μm. **D**. RT-PCR assay was used to measure the relative abundance of mBFP mRNA in tobacco leaves which transiently expressed pBin-R-Er-mBFP (Lanes 1, 3 and 5) or pBin-R-Er-mBFP-mS_1_C (Lanes 2, 4 and 6) constructs. The *Nt* EF-1α was used as a control. Three independent experiments were carried out. M, 100 bp marker. **E**. The relative abundance of mBFP mRNA to that of *Nt* EF-1α was quantitated by Image J. mBFP, pBin-R-Er-mBFP construct; mBFP-mS_1_C, pBin-R-Er-mBFP-mS_1_C construct.

To explore the potential application of BFPvv variants (mBFP) in plants, at least 16 plant expression vectors were constructed in which expression of mBFP or mBFP-mS_1_C fusion constructs were driven by any one of the three promoters (*RbcS*, *Adh* and DR5) and the expressed proteins were targeted to five different subcellular compartments such as cytosol, apoplast, ER, chloroplast, or mitochondria, respectively (Figure 1). The mBFPvvD8 is an improved variant of BFPvv with mutagenesis in 11 amino acids (Kao et al. [2011]). More recently, mBFPvvD9 which can emit at least 30% brighter blue fluorescence than mBFPvvD8 in *E. coli*, was identified, and carried a point mutation at Arg^199^. To compare the brightness of blue fluorescence between two different variants of mBFP in plant cells, the mBFPvvD8 and mBFPvvD9 were simultaneously and transiently expressed in opposite counterpart of the same tobacco leaves by agroinfiltration. In addition, the expressed mBFP variant proteins were targeted to cytosol (pBin-R-Cy-mBFP), apoplast (pBin-R-Se-mBFP) and mitochondria (pBin-R-Mt-mBFP), respectively (Figure 1). Our results showed that the mBFPvvD9 emitted much brighter blue light in all examined tissues than the mBFPvvD8 did (Figure 2A). Therefore, the mBFPvvD9 was further used in the following experiments.

### Transiently expressed mBFP or mBFP-mS_1_C fusion proteins in tobacco leaves

To study whether mBFP is a useful reporter in leaf tissues, the mBFP gene expression driven by *RbcS* promoter was transiently expressed in tobacco leaves by agroinfiltration, and the expressed mBFP proteins were targeted to five different subcellular compartments (Figure 1). The results showed that the expressed mBFP proteins no matter targeted to which compartments all can emit blue fluorescence under UV excitation as compared to a control (Figure 2B), but that stronger blue fluorescence was exhibited in the cytosol, apoplast and ER (Figure 2B). To test the effect of mBFP fusion protein on blue light emission, the mBFP was fused with the C-terminal region (176 amino acids) of ARV σC protein (Figure 1), which the C-terminal domain of σC protein was protease-stable and responsible for the cellular receptor binding (Calvo et al. [2005]). Then the ER-targeted mBFP or mBFP-mS_1_C fusion protein was simultaneously and transiently expressed in the opposite counterpart of same tobacco leaves by agroinfiltration (Figure 2B). The results showed that mBFP-mS_1_C fusion protein can emit much brighter blue fluorescence than mBFP protein can in leaf tissues (Figure 2B) or in protoplasts (Figure 2C). Since both expression constructs were driven by the same promoter, posttranscriptional regulation such as mRNA or protein stability could possibly be responsible for the difference. Therefore, RT-PCR assay was carried out to investigate the relative abundance of mRNA, and the results showed that plants transiently expressed mBFP-mS_1_C fusion construct could accumulate about 1.8 fold more mRNA than that of non-fusion construct (Figure 2D and E). These results suggested that increasing mBFP mRNA stability could be a future strategy for increasing its expression level. Indeed, several potential cryptic introns and mRNA instability elements were present in the mBFP sequence; for instance, AGGAAGT, GTGGACGT, and GTGAACGT, the homologues of intron splicing donor sequences (AGGTAAGT or GTGTACGT), and ATTTG, the homologue of mRNA instability element (ATTTA), were found. In previous research, modifications of GFP by optimization of codon usage and elimination of a cryptic intron can greatly enhance the GFP expression in plants (Chiu et al. [1996]; Haseloff et al. [1997]). Therefore, to further improve the expression level of mBFP in plants, the codon usage might need to be optimized for plants in the future.

### mBFP is a useful reporter under hypoxic stress or auxin treatment

**Figure 3:**
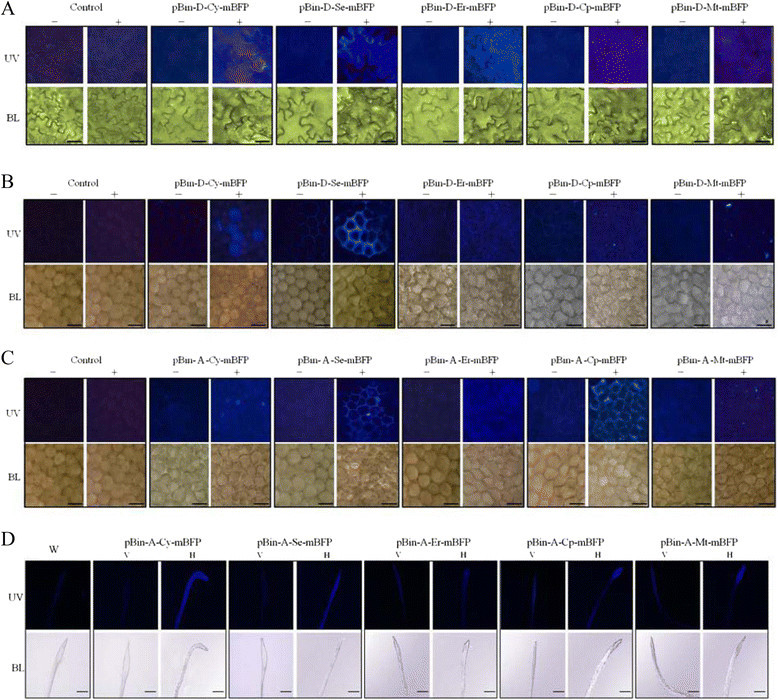
**mBFP was induced as a reporter in different tissue types among plant species under various conditions. A**. Tobacco leaves was infiltrated with Agrobacteria carrying mBFP expression constructs driven by DR5 promoter. The infiltrated tissues were further treated with or without 50 μM NAA for 24 hr. Subsequently, the tissues were imaged under fluorescence microscope with UV excitation (UV) or bright light (BL). The induced mBFP proteins were targeted to cytosol (pBin-D-Cy-mBFP), apoplast (pBin-D-Se-mBFP), ER (pBin-D-Er-mBFP), chloroplast (pBin-D-Cp-mBFP) or mitochondria (pBin-D-Mt-mBFP), respectively. Control, *Agrobacteria* only; +, 50 μM NAA treatment; −, mock treatment. **B**. The flowers of moth orchid were carried out as the same as described in **A**. **C**. The mBFP expression cassettes driven by *Adh* promoter were transiently expressed in the petals of moth orchid. After agroinfiltration, the inflorescences were treated with or without 1 mM H_2_O_2_ for 24 hr. The induced mBFP proteins were targeted to cytosol (pBin-A-Cy-mBFP), apoplast (pBin-A-Se-mBFP), ER (pBin-A-Er-mBFP), chloroplast (pBin-A-Cp-mBFP) or mitochondria (pBin-A-Mt-mBFP), respectively. Control, *Agrobacteria* only; +, 1 mM H_2_O_2_ treatment; −, mock treatment. **D**. Five different transgenic Arabidopsis lines in which mBFP gene expression was driven by *Adh* promoter were generated by floral dip method, and the expressed proteins were targeted to cytosol (pBin-A-Cy-mBFP line), apoplast (pBin-A-Se-mBFP line), ER (pBin-A-Er-mBFP line), chloroplast (pBin-A-Cp-mBFP line) or mitochondria (pBin-A-Mt-mBFP line), respectively. The seeds of Arabidopsis were plated on MS media containing kanamycin, and subsequently were grown vertically (V) or horizontally (H) for 14 days after germination. The regions surrounding the root tips were imaged under fluorescence microscope with UV excitation (UV) or bright light (BL). W, wild type Arabidopsis. The magnification is 400 X **(A)**, 200 X **(B, C)** or 40 X **(D)** and the ruler is 20 μm **(A)**, 40 μm **(B, C)** or 1 mm **(D)**.

Further experiments were conducted to investigate whether or not the mBFP could be induced and imaged in different subcellular compartments and tissue types among plant species under various conditions. Firstly, five mBFP expression vectors driven by synthetic auxin-inducible DR5 promoter were constructed, and the expressed proteins were targeted to five different subcellular compartments (Figure 1). After agroinfiltration of tobacco leaves and flowers of moth orchid, they were then treated with or without 50 μM NAA for 24 hr. The results showed that mBFP blue fluorescence could be imaged in five subcellular compartments of tobacco leaf tissues and the petals of moth orchid, but that it showed brighter blue fluorescence in the cytosol, apoplast, and ER (Figure 3A and B). Secondly, five mBFP expression vectors driven by hypoxia-inducible *Adh* promoter were constructed, and the expressed proteins were targeted to five different subcellular compartments (Figure 1). Previously, it has been shown that H_2_O_2_ levels is significantly increased after O_2_ deprivation and is required to trigger *Adh* gene expression (Baxter-Burrell et al. [2002]). Therefore, after agroinfiltration, the inflorescences of moth orchid were treated with or without 1 mM H_2_O_2_ for 24 hr. The results showed that although the mBFP blue fluorescence could be observed in all five subcellular compartments, the cytosol, apoplast, ER and chloroplasts showed brighter blue fluorescence (Figure 3C). Previously, it has been reported that the roots of horizontally rather than vertically grown Arabidopsis will be under hypoxia pressure upon growing into the gel of a medium plate (Chung and Ferl [1999]). To investigate if mBFP was suitable for using as a reporter under hypoxic conditions, five transgenic Arabidopsis lines in which the mBFP expression was driven by *Adh* promoter, along with target proteins expected to accumulate in five different subcellular compartments, were generated. When the transgenic Arabidopsis lines were grown horizontally, the mBFP expression could be induced and the blue fluorescence could be clearly imaged in the root tips of transgenic pBin-A-Cy-mBFP (mBFP accumulated in cytosol) line, pBin-A-Se-mBFP (apoplast) line, pBin-A-Cp-mBFP (chloroplast) line and pBin-A-Mt-mBFP (mitochondria) line as compared with that in corresponding vertically grown plants (Figure 3D). These results suggested that mBFP is a useful reporter under hypoxic environments.

### Effect of NADPH on intensity of mBFP blue fluorescence

**Figure 4:**
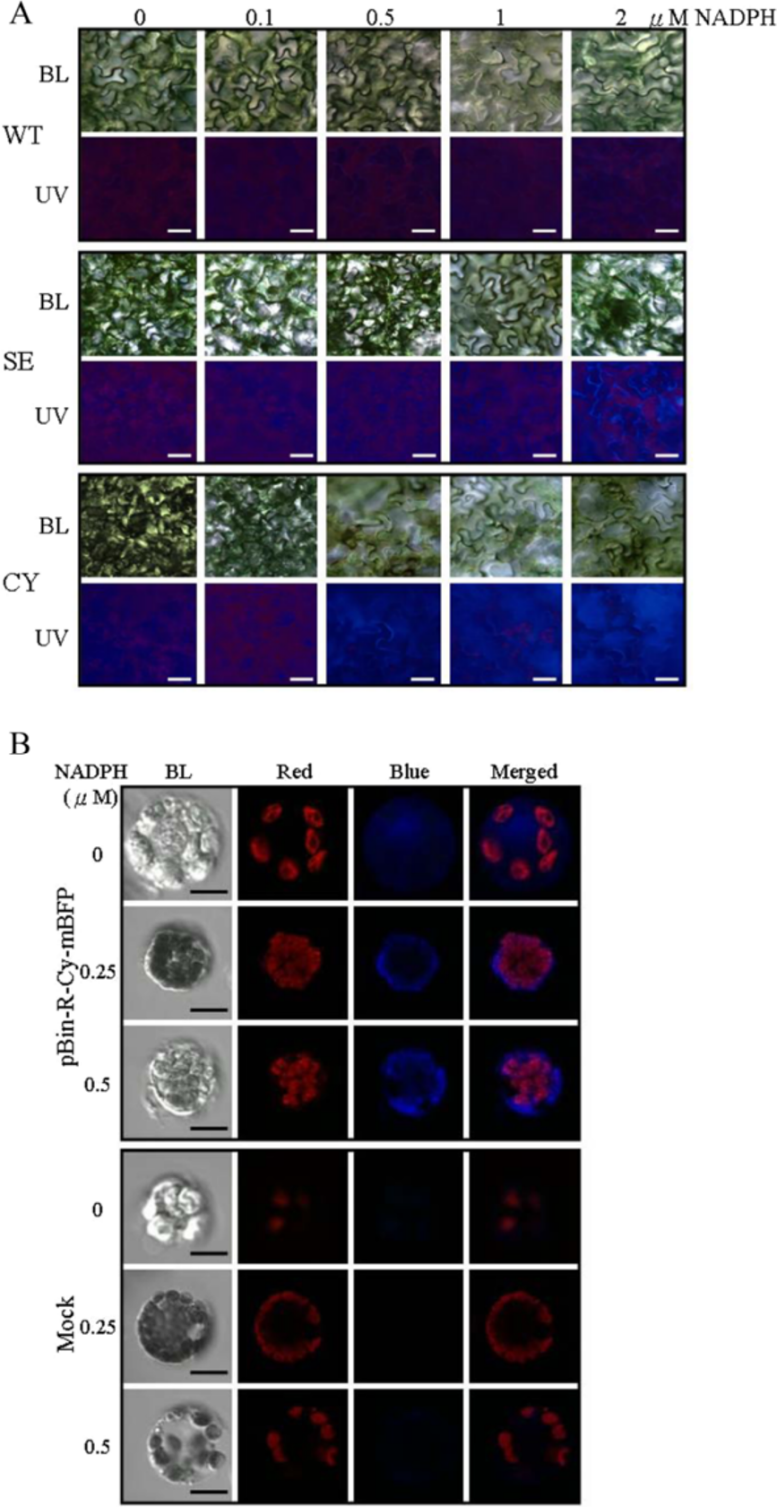
**Effects of NADPH on the brightness of mBFP blue fluorescence. A**. The leaves of wild type (WT) and transgenic Arabidopsis pBin-R-Se-mBFP (SE) line or pBin-A-Cy-mBFP (CY) line were directly infiltrated with NADPH as indicated concentration. Then the leaf tissues were imaged under fluorescence microscope with UV excitation (UV) or bright light (BL). The magnification is 400 X and the ruler is 20 μm. **B**. Tobacco leaves were infiltrated with Agrobacteria carrying pBin-R-Cy-mBFP construct or Agrobacteria only (Mock). The protoplasts isolated from infiltrated leaves were treated with NADPH, and subsequently were imaged under confocal microscope. BL, bright light. Red, chlorophyll auto-fluorescence; Blue, mBFP blue fluorescence. The magnification is 1,000 X and the ruler is 20 μm.

NADPH is essential for mBFP blue fluorescence under UV excitation because the mechanism of mBFP blue fluorescence emission is due to the augmentation of the intrinsic bound NADPH fluorescence (Chang et al. [2004b]; Kao et al. [2011]). To investigate the effect of NADPH concentration on mBFP blue fluorescence *in planta*, leaf tissues of wild type or transgenic Arabidopsis pBin-R-Cy-mBFP and pBin-R-Se-mBFP lines in which the expressed mBFP protein was accumulated in cytosol and apoplast, respectively, were infiltrated with different NADPH concentrations (0, 0.1, 0.5, 1 and 2 μM) before imaging. The results showed that the higher the NADPH concentration, the stronger the mBFP blue fluorescence was emitted under UV excitation (Figure 4A). Alternatively, leaf disks of transgenic Arabidopsis (pBin-R-Se-mBFP line) were incubated with different concentrations of NADPH (0, 0.1, 0.5, 1 and 2 μM) for 15 min under suction before imaging. This result is consistent that mBFP blue fluorescence was significantly amplified with increasing NADPH concentrations (Additional file 2: Figure S1). Furthermore, when the protoplasts isolated from tobacco leaves which mBFP was transiently expressed and targeted to cytosol were treated with different NADPH concentrations (0, 0.25 and 0.5 μM), brighter mBFP blue fluorescence was imaged in the presence of higher amounts of NADPH under confocal microscope analysis (Figure 4B). Although NADPH itself displays fluorescence which may interfere with the emission of fluorescence from mBFP-bounded NADPH, fluorescence from the free form of NADPH was not detectable under 0.5 μM NADPH (Figure 4B).

Applications of the fluorescent proteins as biosensors are very useful for quantitative live imaging (Okumoto et al. [2012]). Previously, it has been shown that mBFPvvD8 could be applied in a Förster resonance energy transfer (FRET) assay (Kao et al. [2011]). Although the NADPH participates in many oxido-reductive reactions, and might be universally present in plant cells, its concentration may vary significantly in different subcellular compartments and tissue types. In this study, the brightness of mBFP fluorescence was shown to be positively correlated with the NADPH concentration (Figure 4). The results suggested that the mBFP might have the potential to be further developed as an NADPH biosensor *in planta*.

### Subcellular localization of mBFP by confocal microscope analysis

**Figure 5:**
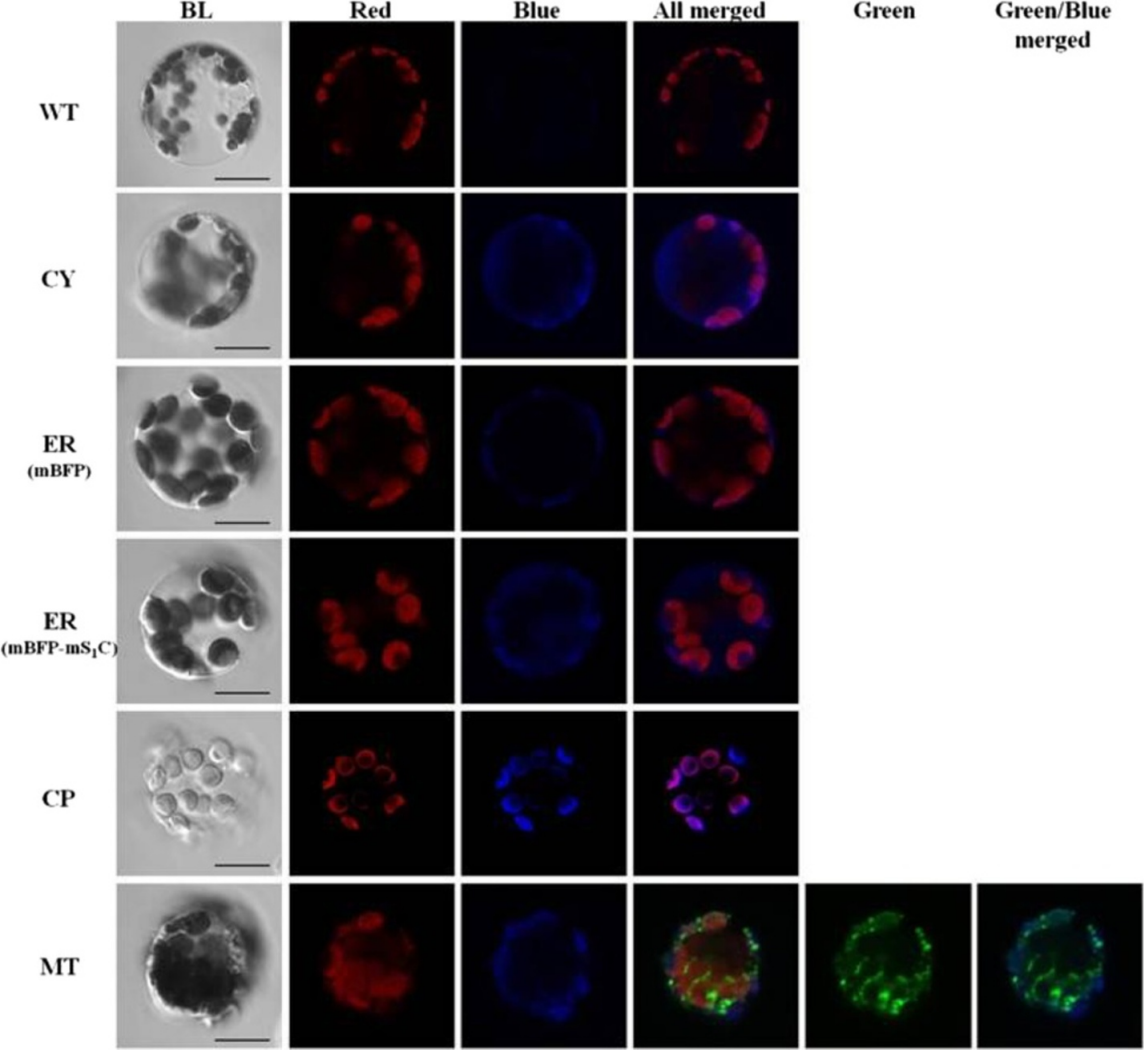
**Subcellular localization of mBFP or mBFP-mS**_**1**_**C fusion protein in protoplasts of transgenic Arabidopsis.** Five different transgenic Arabidopsis lines in which the expression of mBFP or mBFP-mS_1_C gene was driven by *RbcS* promoter were generated by floral dip method, and the expressed mBFP or mBFP-mS_1_C fusion proteins were expected to accumulate in the cytosol (CY, pBin-R-Cy-mBFP), ER (mBFP, pBin-R-Er-mBFP; mBFP-mS_1_C, pBin-R-Er-mBFP-mS_1_C), chloroplast (CP, pBin-R-Cp-mBFP) or mitochondria (MT, pBin-R-Mt-mBFP), respectively. The protoplasts were isolated from the leaf tissues of transgenic Arabidopsis lines and wild type (WT), and then were imaged under confocal microscope. Alternatively, the protoplasts isolated from transgenic pBin-R-Mt-mBFP line were treated with MitoTracker (Invitrogen) before imaging. BL, bright light; Red, chlorophyll auto-fluorescence; Blue, mBFP blue fluorescence; Green, green fluorescence of MitoTracker. The magnification is 1,000 X and the ruler shown is 20 μm in length.

Previous study have shown that signal peptide which helps to direct the target protein into subcellular compartments may be masked by the target protein itself, and thus causes mis-targeting or multiple targeting of the protein (Tanz et al. [2013]). In this study, mBFP accumulation in apoplast was obvious in the tested tissues (Figures 2, 3 and 4). To investigate if mBFP was correctly targeted into other compartments, the protoplasts were isolated from leaves of five transgenic Arabidopsis lines in which mBFP proteins were expected to accumulate in cytosol (pBin-R-Cy-mBFP line), ER (pBin-R-Er-mBFP and pBin-R-Er-mBFP-mS_1_C lines), chloroplast (pBin-R-Cp-mBFP line) and mitochondria (pBin-R-Mt-mBFP line), respectively, and were imaged under confocal microscope. The ER-resident protein used the same secretory pathway as that of protein targeted to apoplast, except for having an additional ER-retention signal in the C-terminal. Although no ER-specific marker was used in this study, the mBFP or mBFP-mS_1_C protein was seen to be intracellularly present, suggesting that it was ER-localized (Figures 2C and 5). The mBFP targeted to chloroplast was confirmed because of co-localization of mBFP blue fluorescence with chlorophyll auto-fluorescence (Figure 5). Therefore, our result showed that mBFP and mBFP-mS_1_C fusion proteins were accumulated in the correct subcellular compartments except mitochondria (Figure 5), because the green fluorescence of MitoTracker was only partially co-localized with mBFP blue fluorescence in the protoplasts isolated from transgenic Arabidopsis (pBin-R-Mt-mBFP line) (Figure 5). Therefore, the protoplasts were isolated from tobacco leaves which transiently expressed pBin-R-Mt-mBFP construct for further investigation. The result was consistent with above that mBFP is partially co-localized with Mitotracker (Additional file 3: Figure S2). It suggested that the mitochondria-targeted signal peptide might have been masked by mBFP, thus making it unable to be imported or inefficiently imported into mitochondria. Therefore, it will be better to avoid fusing mBFP in the N-terminal when apply a fusion mBFP tag protein for importing to plant mitochondria in future applications.

## Conclusions

Through expression of mBFP or mBFP-mS_1_C fusion protein in different subcellular compartments and tissue types among three different plant species under various conditions, in this study, we demonstrated that mBFP protein from *Vibrio vulnificus* CKM-1 is a useful reporter *in planta*. It could be used, for example, as a fusion tag to study protein trafficking and localization under both aerobic and anaerobic environments. In addition, the brightness of mBFP blue fluorescence was positively correlated to the NADPH concentration which might suggest that mBFP has great potential to be further developed as a NADPH biosensor for plant research. Although the blue auto-fluorescence coming from secondary cell wall might limit the utility of blue fluorescence protein in plant research, however, when the experiments were carried out with strict control, it will be applicable and useful as a reporter as shown in this study. Furthermore, further improvement of mBFP through codon optimization for plants might significantly enhance its brightness and expand its potential applications.

## Additional files

## Electronic supplementary material

Additional file 1: Table S1.: The PCR primers used in this study. (PDF 52 KB)

Additional file 2: Figure S1.: Effects of NADPH on the brightness of mBFP blue fluorescence in leaf disks of transgenic Arabidopsis. (PDF 103 KB)

Additional file 3: Figure S2.: Subcellular localization of mBFP in protoplasts of transiently expressed tobacco leaves. (PDF 96 KB)

Below are the links to the authors’ original submitted files for images.

Authors’ original file for figure 1

Authors’ original file for figure 2

Authors’ original file for figure 3

Authors’ original file for figure 4

Authors’ original file for figure 5

## Abbreviations

ARV: Avian reovirus
Adh: Alcohol dehydrogenase gene
BFPs: Blue fluorescent proteins
BFPvv: NADPH-dependent blue fluorescent protein
DR5: Synthetic auxin-inducible promoter
ER: Endoplasmic reticulum
GFP: Green fluorescent protein
mBFP: Variant of blue fluorescent protein BFPvv
*Nt* EF-1α: Tobacco elongation factor 1α gene
Nos: Nopaline synthase gene
RbcS: Ribulose 1, 5-bisphosphate carboxylase/oxygenase small subunit gene
