# Supplementary material for: The blue fluorescent protein from Vibrio vulnificus CKM-1 is a useful reporter for plant research
Source: Bot Stud. 2014 Dec 17;55:79. doi: 10.1186/s40529-014-0079-x (PMC5432841; doi:10.1186/s40529-014-0079-x)
Supplement: Supplementary file 1 — Additional file 1: Table S1.: The PCR primers used in this study. (PDF 52 KB) [file 40529_2014_9079_MOESM1_ESM.pdf]

Supplemental Table

Supplemental Table 1 The PCR primers used in this study.

Primer name	Sequence[a] (5'-3')	Purpose
NcoI-mBFP-F	ACCATGGGAAAGAAACTGGTGGTCATCAC	PCR cloning of mBFP gene into ImpactVectors
SacI-mBFP$_{1245}$-R	AGAGCTCAAGGCTGCTGCTTTGTGGGAG	
NotI-mBFP$_{3}$-R	AGCGGCCGCAGGCTGCTGCTTTGTGGGAG	
XbaI-Tp-F	GATCTAGAATGGCCTCGATCTCTTCCTCCGCT	Construction of mBFP expression cassette for targeting to chloroplast
SacI-mBFP$_{1245}$-R	AGAGCTCAAGGCTGCTGCTTTGTGGGAG	
HindIII-DR5-F	AAAAGCTTCCTTTTGTCTCC	PCR amplification of DR5 promoter for cloning
XbaI-DR5-R	ATCTAGAGGTGGACTCCTCTTAATTGTA	
NotI-mS$_1$C-F	AGCGGCCGCATCATTGGAGTCTACCGCT	Construction of mBFP-mS$_1$C fusion protein gene
BglII-mS$_1$C-R	TAGATCTGGTGTCGATGCCGGTACG	
HindIII-AdhP-F	CCAAGCTTGAATAATACTATTAAAGAGAGC	PCR amplification of *Adh* promoter from Arabidopsis genome
NcoI-AdhP-R	CCCATGGTTTGTTAGTTTTGTGTGATTGTGATG	
EF-1α-F	GGTAGGATACAACCCTGATAAAATC	PCR amplification of tobacco EF-1α cDNA
EF-1α-R	CAGTGGGACCAAAAGTCACAAC	

[a]The sequence underline is the restriction enzyme sites.
