# Supplementary material for: The blue fluorescent protein from Vibrio vulnificus CKM-1 is a useful reporter for plant research
Source: Bot Stud. 2014 Dec 17;55:79. doi: 10.1186/s40529-014-0079-x (PMC5432841; doi:10.1186/s40529-014-0079-x)
Supplement: Supplementary file 2 — Additional file 2: Figure S1.: Effects of NADPH on the brightness of mBFP blue fluorescence in leaf disks of transgenic Arabidopsis. (PDF 103 KB) [file 40529_2014_9079_MOESM2_ESM.pdf]

Supplemental Figures

Supplemental Fig. 1

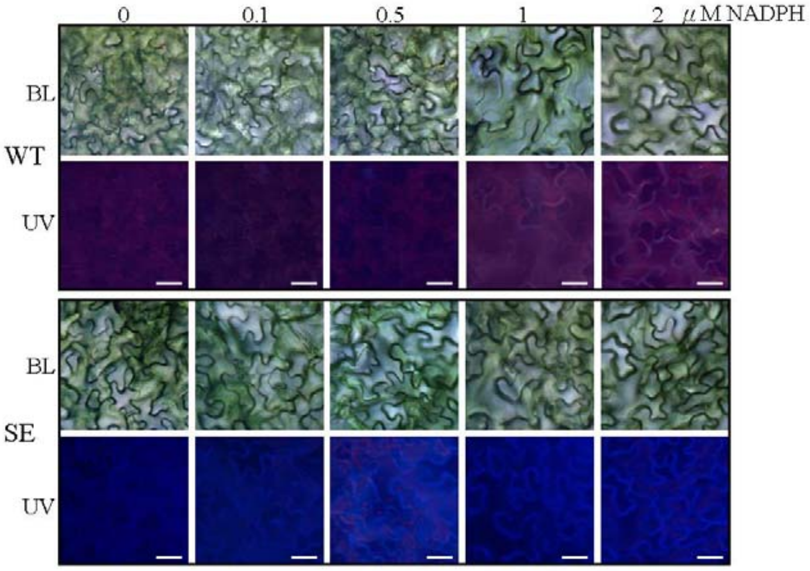

Supplemental Fig. 1 Effects of NADPH on the brightness of mBFP blue fluorescence in leaf disks of transgenic Arabidopsis.

The leaf disks of transgenic Arabidopsis pBin-R-Se-mBFP (SE) line and wild type Arabidopsis (WT) were treated with different concentrations of NADPH as indicated under vacuum for 15 min. Then the leaf tissues were imaged under fluorescence microscope with UV excitation (UV) or bright light (BL). The magnification is 400 X and the ruler shown is 20 μm in length.
