# Supplementary material for: The blue fluorescent protein from Vibrio vulnificus CKM-1 is a useful reporter for plant research
Source: Bot Stud. 2014 Dec 17;55:79. doi: 10.1186/s40529-014-0079-x (PMC5432841; doi:10.1186/s40529-014-0079-x)
Supplement: Supplementary file 3 — Additional file 3: Figure S2.: Subcellular localization of mBFP in protoplasts of transiently expressed tobacco leaves. (PDF 96 KB) [file 40529_2014_9079_MOESM3_ESM.pdf]

Supplemental Fig. 2

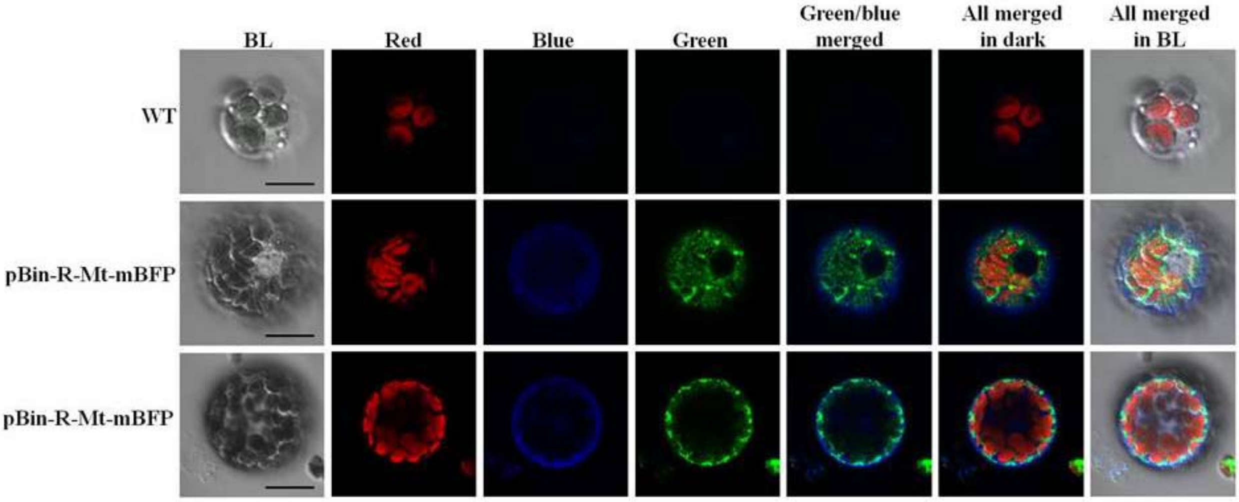

Supplemental Fig. 2 Subcellular localization of mBFP in protoplasts of transiently expressed tobacco leaves.

The mBFP (pBin-R-Mt-mBFP construct) gene was transiently expressed in tobacco leaves by agroinfiltration. The protoplasts isolated from the infiltrated leaf tissues were treated with MitoTracker, and the subcellular localization of mBFP was imaged under confocal microscope. Two different protoplasts (pBin-R-Mt-mBFP) isolated from Agrobacteria infiltrated tobacco leaves were shown. WT, protoplast from mock infiltration (Agrobacteria only); BL, bright light; Red, chlorophyll auto-fluorescence; Blue, mBFP blue fluorescence; Green, green fluorescence of MitoTracker. All merged in dark or in BL indicated the merged picture of red, blue and green fluorescence in dark or bright field, respectively. The magnification is 1,000 X and the ruler shown is 20 μm in length.
