# Supplementary figures and images for: The blue fluorescent protein from Vibrio vulnificus CKM-1 is a useful reporter for plant research
Source: Bot Stud. 2014 Dec 17;55:79. doi: 10.1186/s40529-014-0079-x (PMC5432841; doi:10.1186/s40529-014-0079-x)

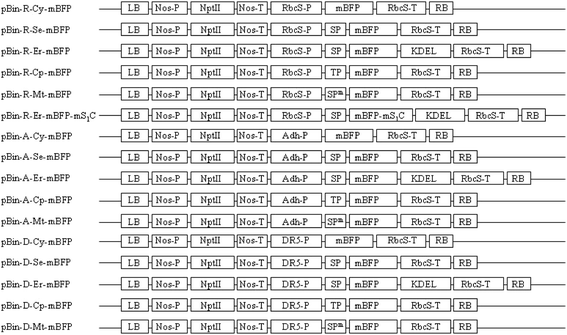

Supplement: Supplementary file 4 — Authors’ original file for figure 1 [file 40529_2014_9079_MOESM4_ESM.gif]

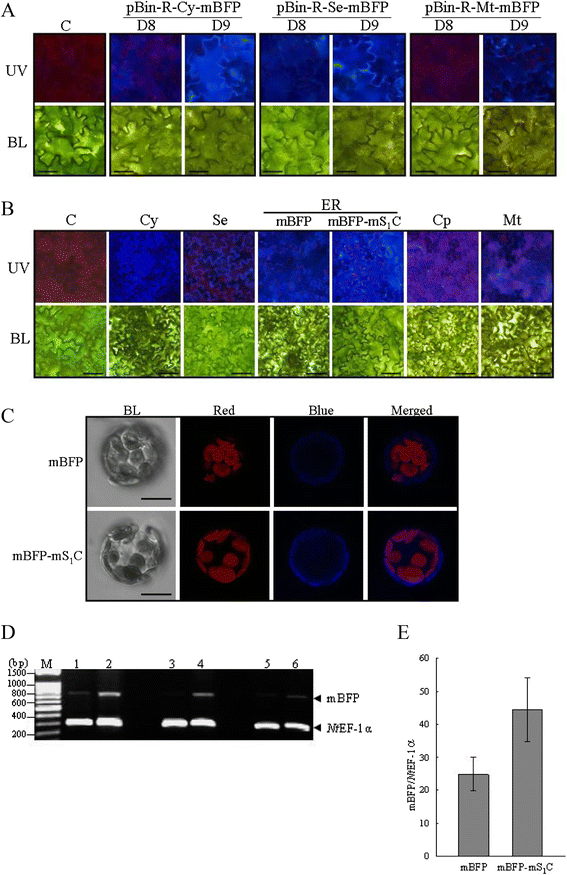

Supplement: Supplementary file 5 — Authors’ original file for figure 2 [file 40529_2014_9079_MOESM5_ESM.gif]

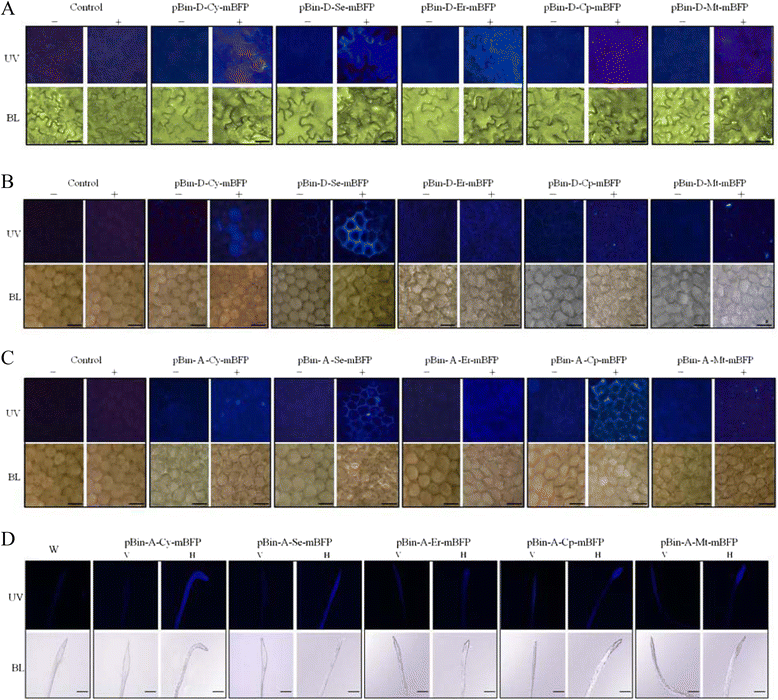

Supplement: Supplementary file 6 — Authors’ original file for figure 3 [file 40529_2014_9079_MOESM6_ESM.gif]

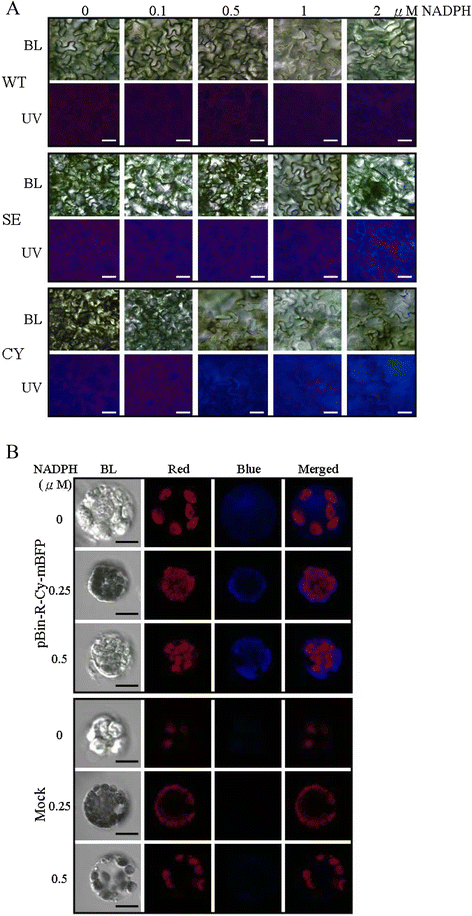

Supplement: Supplementary file 7 — Authors’ original file for figure 4 [file 40529_2014_9079_MOESM7_ESM.gif]

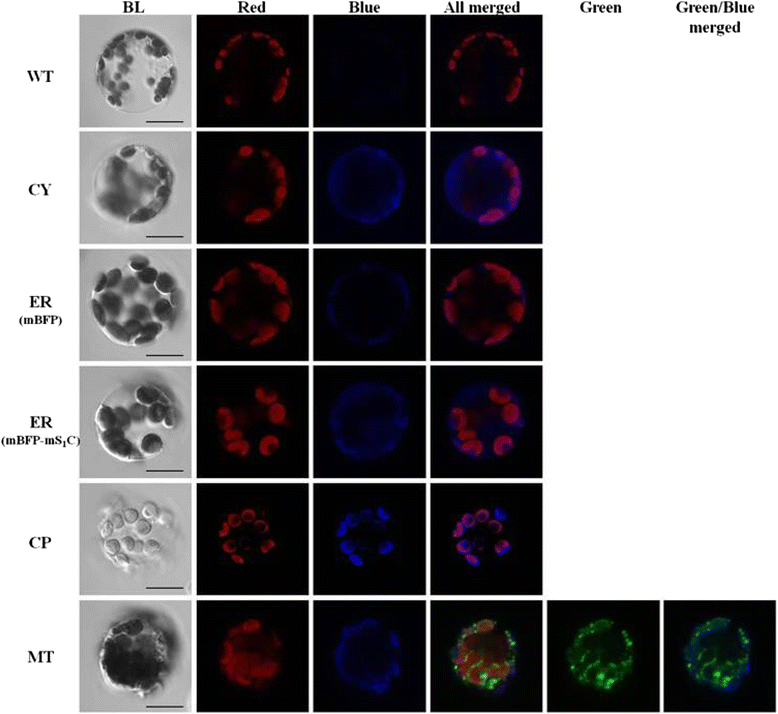

Supplement: Supplementary file 8 — Authors’ original file for figure 5 [file 40529_2014_9079_MOESM8_ESM.gif]
